# Septin4 Prevents PDGF-BB-induced HAVSMC Phenotypic Transformation, Proliferation and Migration by Promoting SIRT1-STAT3 Deacetylation and Dephosphorylation

**DOI:** 10.7150/ijbs.39843

**Published:** 2020-01-14

**Authors:** Naijin Zhang, Ying Zhang, Shilong You, Yichen Tian, Saien Lu, Liu Cao, Yingxian Sun

**Affiliations:** 1 Department of Cardiology, the First Hospital of China Medical University, Shenyang, Liaoning, China.; 2 Key Laboratory of Medical Cell Biology, Ministry of Education; Institute of Translational Medicine, China Medical University; Liaoning Province Collaborative Innovation Center of Aging Related Disease Diagnosis and Treatment and Prevention, Shenyang, Liaoning, China.

**Keywords:** Septin4, STAT3, SIRT1, Atherosclerosis.

## Abstract

SIRT1 and STAT3 are key to human aortic vascular smooth muscle cells (HAVSMCs) proliferation, migration and phenotypic transformation, but the regulatory mechanism of SIRT1-STAT3 in this process is still unclear. Septin4 is a cytoskeleton-related protein that regulates oxidative stress-vascular endothelial injury. However, the role and underlying mechanism of Septin4 in atherosclerosis remains unknown. Here, we revealed the role and mechanism of Septin4 in regulating SIRT1-STAT3 in atherosclerosis. We determined that the expression of Septin4 were markedly increased in Apoe^-/-^ atherosclerosis mice and PDGF-BB-induced HAVSMCs. Knockdown of Septin4 significantly increased PDGF-BB-induced HAVSMCs proliferation, migration and phenotypic transformation, while overexpression of Septin4 had the opposite effects. Mechanically, co-immunoprecipitation results demonstrated that Septin4 was a novel interacting protein of STAT3 and SIRT1. Septin4 formed a complex with SIRT1-STAT3, enhancing the interaction between SIRT1 and STAT3, ensuing promoting SIRT1-regulated STAT3-K685 deacetylation and STAT3-Y705 dephosphorylation, which inhibited PDGF-BB-induced HAVSMCs proliferation, migration and phenotype transformation. Therefore, our findings provide novel insights into the prevention and treatment of atherosclerosis.

## Introduction

Despite the improvement of treatment methods, the incidence and mortality of atherosclerosis are still increasing 1. Proliferation and migration of human aortic vascular smooth muscle cells (HAVSMCs) are the core factors leading to atherosclerosis 2. According to the physiological function of HAVSMCs, HAVSMCs contain synthetic and contractile phenotypes. In mature and normal blood vessels, HAVSMCs are characterized to be contractile phenotype, maintaining vascular tension 3-4. When HAVSMCs are stimulated by various cytokines, such as platelet-derived growth factor BB (PDGF-BB), HAVSMCs will change from contractile phenotype to synthetic phenotype, which enhances the proliferation and migration of HAVSMCs, ensuring inducing atherosclerosis 5-6.

The constant recognition of the influence of proteins post-translational modification on the HAVSMCs has opened up new horizons of studies involving in atherosclerosis. Septin4 is a member of GTP-binding proteins, belonging to the Septins family genes that are essential for cell separation, apoptosis, vesicle trafficking, tumor suppression and other cell processes 7-11. Septin4 has long been considered as an apoptotic and injurious protein. Our previous study has shown that Septin4 can promote oxidative stress-induced vascular endothelial cells damage by interacting with poly ADP-ribose polymerase 1 (PARP1) 12. However, whether Septin4 is involved in phenotypic transformation, proliferation and migration of HAVSMCs and atherosclerosis has not been reported.

Signal transducer and activator of transcription 3 (STAT3) is a transcription activator, which enters the nucleus and binds to promoter sequences of target genes to promote their transcription 13-18. STAT3 plays an important role in phenotype transformation, proliferation and migration of HAVSMCs 3, 13-20. The phosphorylation of STAT3 is the key to promote the proliferation and migration of HAVSMCs 16-18. Previous studies have shown that the tyr phosphorylation of STAT3 was regulated by SIRT1, which deacetylation modified STAT3, ensuing reducing its phosphorylation level 19-20. Therefore, SIRT1 is considered to be a key protein against phenotype transformation, proliferation and migration of HAVSMCs 21-22. However, the regulatory mechanism of SIRT1-STAT3 in this process is still unclear.

Here, we firstly showed that Septin4 is significantly increased during the development of atherosclerosis in Apoe^-/-^ mice, and PDGF-BB-induced proliferation, migration and phenotypic transformation in HAVSMCs. Septin4 knockdown significantly promoted PDGF-BB-induced proliferation, migration and phenotype transformation of HAVSMCs, while Septin4 overexpression remarkably reduced this phenomenon. Mechanically, co-immunoprecipitation identified that Septin4 is a novel interacting protein of STAT3 and SIRT1, forming a complex with SIRT1-STAT3, ensuing promoting the interaction between SIRT1 and STAT3. In addition, Septin4 promotes SIRT1-regulated STAT3-K685 acetylation and STAT3-Y705 phosphorylation reductions in PDGF-BB-induced HAVSMCs model.

## Materials and Methods

### Mice experiments

Specific pathogen-free (SPF) male ApoE^-/-^ (n=8) and ApoE^+/+^ (n=8) mice (8-10 weeks) were purchased form Vitalriver company and housed in individually ventilated cages with 12 hours light/dark cycle and controlled temperature (20 °C-22 °C). High-fat diet (containing 21% fat and 0.15% cholesterol) for ApoE^-/-^ and ApoE^+/+^ mice were performed for 8 weeks to induce atherosclerosis model (each group of mice, n=8). Hematoxylin and eosin (H&E) staining and Western-blot were performed for mice in each group. Aortic root vascular tissue specimens from mice were fixed with 4% formalin (4h), paraffin embedded and sectioned at 5-µm. After xylene dewaxing and rehydration by graded ethyl alcohol, the sections underwent H&E staining. All animal handling complied with animal welfare regulations of China Medical University. The Animal Subjects Committee of China Medical University approved the animal study protocol. The investigation conforms to the guide for the care and use of laboratory animals published by the US National Institutes of Health (NIH Publication No. 85-23, revised 1985).

### Cell culture and cell transfections

HAVSMCs were provided by Cambrex (China Center for Type Culture Collection, China) and maintained in H-Dulbecco's modified Eagle medium (H-DMEM) (HyClone, USA) containing 10% fetal bovine serum (FBS) (HyClone) in a humid environment with 5% CO_2_ at 37˚C. HAVSMCs were passaged 4-6 times before use. Plasmids encoding the full-length human Septin4 (Shanghai Genechem) was cloned to Flag tagged destination vectors. Transfection was performed with Lipofectamine 3000 (Invitrogen, USA) as directed by the manufacturer. Control- and Septin4-siRNAs were provided by RIBOBIO (China). Septin4 knockdown was performed with the jetPRIME transfection reagent (PolyPlus, France). Three target sequences were assessed for excluding off-target effects. Septin4 knockdown efficiency was confirmed by immunoblot.

Septin4 siRNA-1: GGACCGGAAACTTCTTGGT;

Septin4 siRNA-2: GGAGATCACTAAGCATGCA;

Septin4 siRNA-3: TGGCAGAATACATTGATCA.

### Antibodies and reagents

Antibodies to polyclone rabbit anti-Septin4 (1:1000; abcam, USA; 1:500; Proteintech, USA), monoclonal rabbit anti-Flag (1:1000; Cell Signaling Technology, USA), polyclone rabbit anti-STAT3-K685 (1:1000; Cell Signaling Technology, USA), polyclone rabbit anti-STAT3-Y705 (1:1000; Cell Signaling Technology, USA), polyclonal rabbit anti-STAT3 (1:1000; Proteintech, USA), polyclonal rabbit anti-SIRT1 (1:1000; Proteintech, USA), polyclonal rabbit anti-SM22α (1:1000; Proteintech, USA), polyclonal rabbit anti-α-SM-actin (1:1000; Proteintech, USA), monoclonal rabbit anti-MMP9 (1:200; Santa, USA), polyclonal rabbit anti-MMP2 (1:500; Proteintech, USA), polyclonal rabbit anti-PCNA (1:1000; Proteintech, USA) and monoclonal mouse anti-GAPDH (1:2000; abcam, USA) were purchased commercially. Protein A/G immunoprecipitation magnetic beads was obtained from Biotool and used for immunoprecipitation.

### Co-immunoprecipitation and Western blot

For the purpose of co-immunoprecipitation, the cells were washed twice with a newly prepared protease inhibitor and dissolved with a marker solution buffer. The lysates were incubated with antibody (3 hours) and protein A/G immunoprecipitation beads (Biotool, USA) (12 hours, 4 °C). The immune complex was detected by SDS-PAGE.

Western blot was used co-immunoprecipitation buffer for cell lysis. The protein was extracted by centrifugation (13300 rpm; 20 min, 4 °C). BCA protein detection kit (Dingguo Changsheng Biotechnology, China) was used for total protein quantification. The same amount of protein was separated by SDS = page and transferred to PVDF membrane by electricity. Then, 5% bovine serum albumin (BSA) was sealed in Tris buffer tween (TBST) under environmental conditions (1 hours), and then incubated overnight (4 °C) with antibodies. GAPDH was used as a loading control. Image J v1.46 (National Institutes of Health, USA) was employed for analysis.

### Cell viability, Migration and Phalloidin staining assay

Cell Counting Kit-8 (CCK-8; Biotool, USA) was employed to assess cell viability. HAVSMCs were seeded into a 96-well plate at 3x10^3^ cells/well in H-DMEM containing 10% FBS and underwent transfection with control and Flag-Septin4 plasmids, respectively, or control and Septin4-siRNA, respectively. PDGF-BB administration was used for 24 hours after starvation in serum-free medium for 24 hours, and 100 µl of CCK-8 reagent was added per well for 1.5 h. Absorbance was determined at 450 nm on a Bio-Rad microplate reader (Model 680; Bio-Rad, USA).

Cell migration was assessed in transwell plates (Corning Life Sciences, USA). A total of 5x10^3^ cells were implanted into the upper cavity of the 24 well plate. PDGF-BB administration was used for 24 hours after starvation in serum-free medium for 24 hours. The top chambers were used serum-free H-DMEM, however, the lower ones contained with 10% FBS. After incubation at 37 °C overnight, non-migrating cells were removed with cotton swabs. The migrants were fixed with frozen methanol, stained with crystal violet, and 5 random areas were counted.

Phalloidin (Molecular Probes, USA) staining of HAVSMCs was performed after fixation with 4% formalin (20 min) and permeabilization with 0.5% Triton X-100 (30 min), as directed by the manufacturer. Cell morphology and actin filaments were observed under a fluorescence microscope (Olympus).

### Statistical analysis

Data are mean ± standard deviation (SD). Homogeneity of variance was evaluated by the F test (group pair) or Brown-Forsythe test (multiple groups). The Shapiro-Wilk test was performed for assessing data normality. Student's t-test and Welch t test were employed to assess data of group pairs with normal and skewed distributions, respectively (two groups). ANOVA and indicated non-parametric tests were performed to compare multiple groups. One-way ANOVA and two-way ANOVA were performed for comparing groups for single and two factors, respectively. *P* values were adjusted for multiple comparisons when applicable. All data were analyzed by SPSS 22.0 (SPSS, USA), and *P*<0.05 was considered statistically significant.

## Results

### Septin4 was significantly increased in Apoe^-/-^ atherosclerosis mice and PDGF-BB-induced HAVSMCs

**Figure 1:**
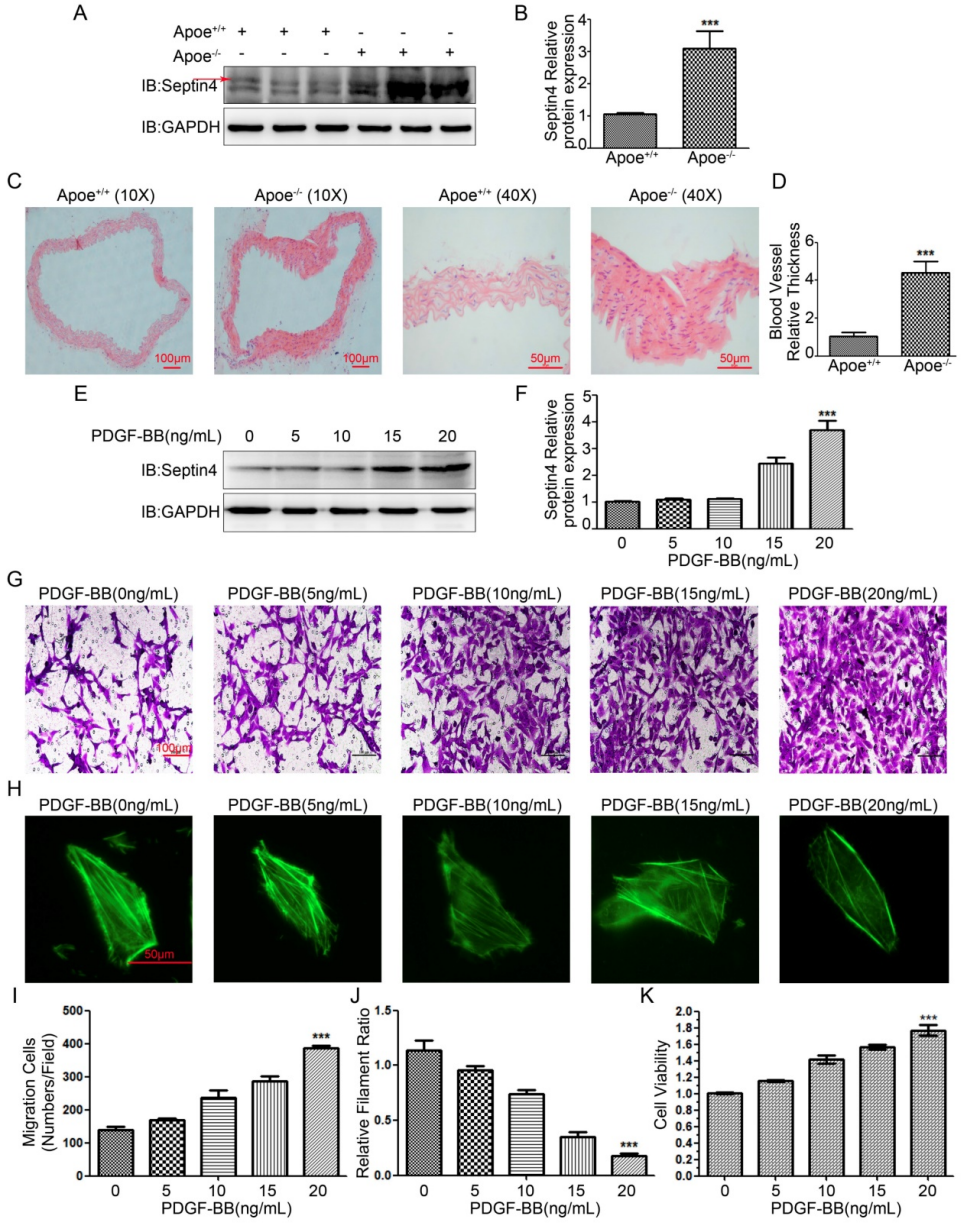
Septin4 was significantly increased in Apoe^-/-^ atherosclerosis mice and PDGF-BB-induced HAVSMCs. **(A)** Septin4 was examined by Western-blot in vascular tissue from Apoe^+/+^ and Apoe^-/-^ mice; **(B)** quantification of results as means ± SD (P<0.001). **(C)** HE staining was measured to assess atherosclerosis in vascular tissue from Apoe^+/+^ and Apoe^-/-^ mice; **(D)** quantification of results as means ± SD (P<0.001). **(E)** Septin4 was examined by Western-blot with a concentration gradient of PDGF-BB; **(F)** quantification of results as means ± SD (P<0.001). **(G)** Transwell was measured to assess the HAVSMCs migration with gradient of PDGF-BB. **(H)** Phalloidine dye was measured to assess the HAVSMCs phenotypic transformation with gradient of PDGF-BB and intracellular myofilaments were labeled with green fluorescence. **(I)** Quantification of Transwell results were shown as means ± SD (P<0.001).** (J)** Quantification of Phalloidine results were shown as means ± SD (P<0.001). **(K)** CCK8 was measured to assess HAVSMCs proliferation with gradient of PDGF-BB and quantification of results as means ± SD (P<0.001).

Firstly, in order to detect Septin4 expression in atherosclerosis model *in vivo,* high-fat diet was used to induce atherosclerosis in Apoe^-/-^ mice. The results showed that compared with Apoe^+/+^ mice, the expression of Septin4 was significantly increased in Apoe^-/-^ mice (*Figure 1A-B*). In addition, we found that the vascular tissues were significantly hypertrophy during the development of atherosclerosis in Apoe^-/-^ mice (*Figure 1C-D*).

Next, in order to detect Septin4 expression *in vitro* model*,* PDGF-BB was used to induce HAVSMCs proliferation, migration and phenotypic transformation. The results showed that with the increase of PDGF-BB concentration, the expression level of Septin4 increased gradually (*Figure 1E-F*). In addition, HAVSMCs had obvious increase in proliferation, migration and phenotypic transformation with the increase of PDGF-BB concentration (*Figure 1G-K*).

*In vivo* and *in vitro* results suggested that Septin4 may be involved in the regulation of atherosclerosis and HAVSMCs proliferation, migration and phenotypic transformation.

### Septin4 significantly inhibited PDGF-BB-induced HAVSMCs proliferation and migration

**Figure 2:**
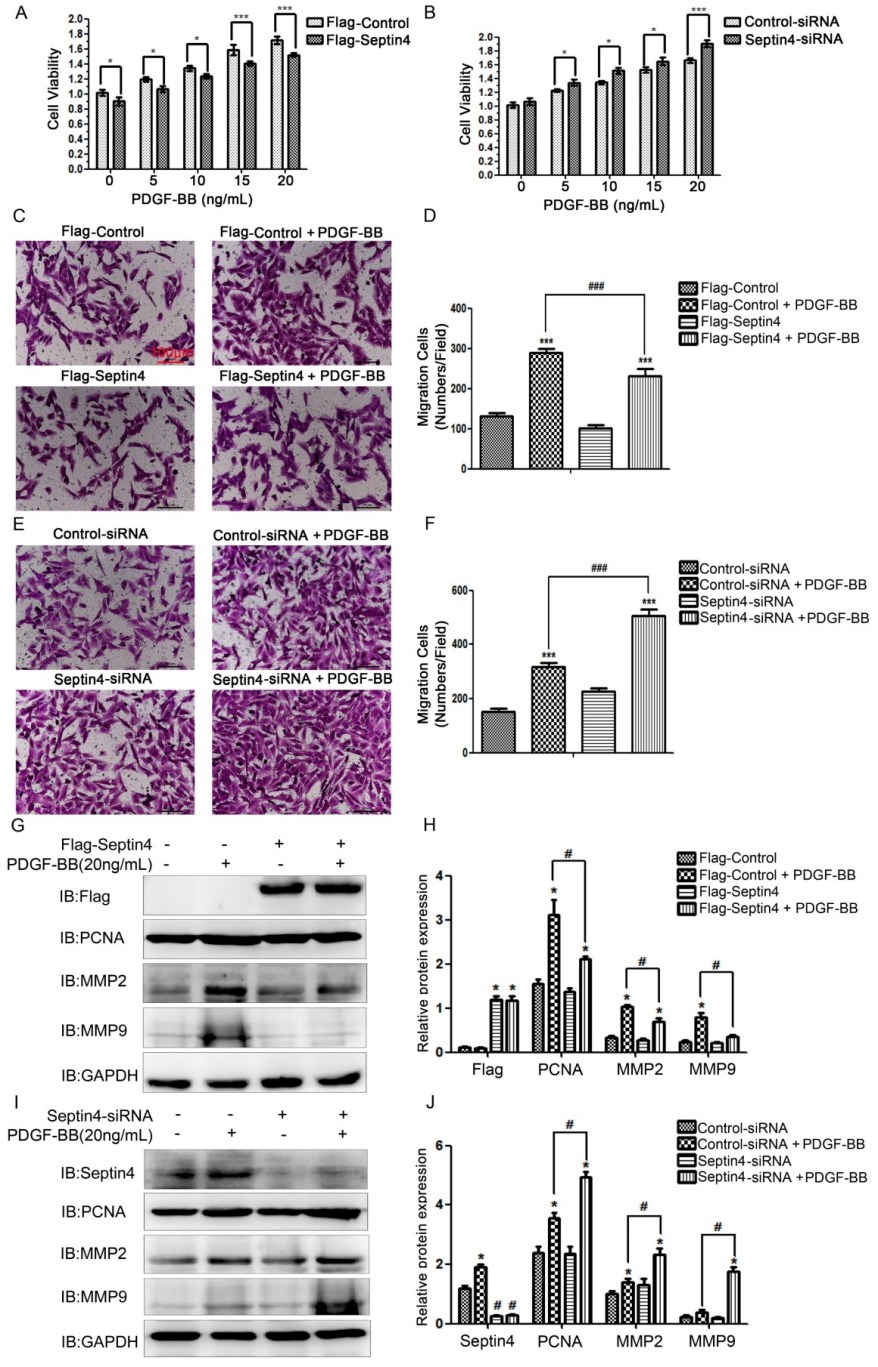
Septin4 inhibited PDGF-BB-induced HAVSMCs proliferation and migration, and downregulates PCNA, MMP2 and MMP9 expression. **(A)** HAVSMCs were transfected with the control or Flag-Septin4 plasmid for 36 hours, and treated with 0, 5, 10 and 20 ng/mL PDGF-BB for 24 hours, respectively. CCK8 was used to assess HAVSMCs viability; quantitated data are mean ± SD (P<0.001). **(B)** As did HAVSMCs were transfected with the control-siRNA or Septin4-siRNA. **(C-D)** HAVSMCs were transfected with the control or Flag-Septin4 plasmid for 36 hours, and treated with or without 20 ng/mL PDGF-BB for 24 hours. Transwell was used to assess HAVSMCs migration; quantitated data are mean ± SD (P<0.001).** (E-F)** As did HAVSMCs were transfected with the control-siRNA or Septin4-siRNA.** (G)** HAVSMCs were transfected with the control or Flag-Septin4 plasmid for 36 hours, and treated with or without 20 ng/mL PDGF-BB for 24 hours and PCNA, MMP2 and MMP9 were detected by Western-blot; **(H)** quantitated data are mean ± SD (P<0.001).** (I-J)** As did HAVSMCs were transfected with the control-siRNA or Septin4-siRNA.

In order to clarify the role of Septin4 in PDGF-BB-induced HAVSMCs proliferation and migration, overexpression and knockdown Septin4 were performed in PDGF-BB-induced HAVSMCs model. CCK8 and transwell experiments showed that overexpression of Septin4 significantly relieved PDGF-BB-induced HAVSMCs proliferation (decreased 11.7%; P<0.001) and migration (decreased 20%; P<0.001) (*Figure 2A, C-D*), while knockdown of Septin4 significantly aggravated PDGF-BB-induced HAVSMCs proliferation (increased 14.5%; P<0.001) and migration (increased 59%; P<0.001) (*Figure 2B, E-F*).

In addition, overexpression of Septin4 significantly decreased PDGF-BB-induced proliferation and migration makers PCNA, MMP2 and MMP9 expression (*Figure 2G-H*). While, knockdown of Septin4 had the opposite effects (*Figure 2I-J*).

These results suggested that Septin4 may be a new regulatory protein against HAVSMCs proliferation and migration.

### Septin4 significantly resisted PDGF-BB-induced HAVSMCs phenotypic transformation

**Figure 3:**
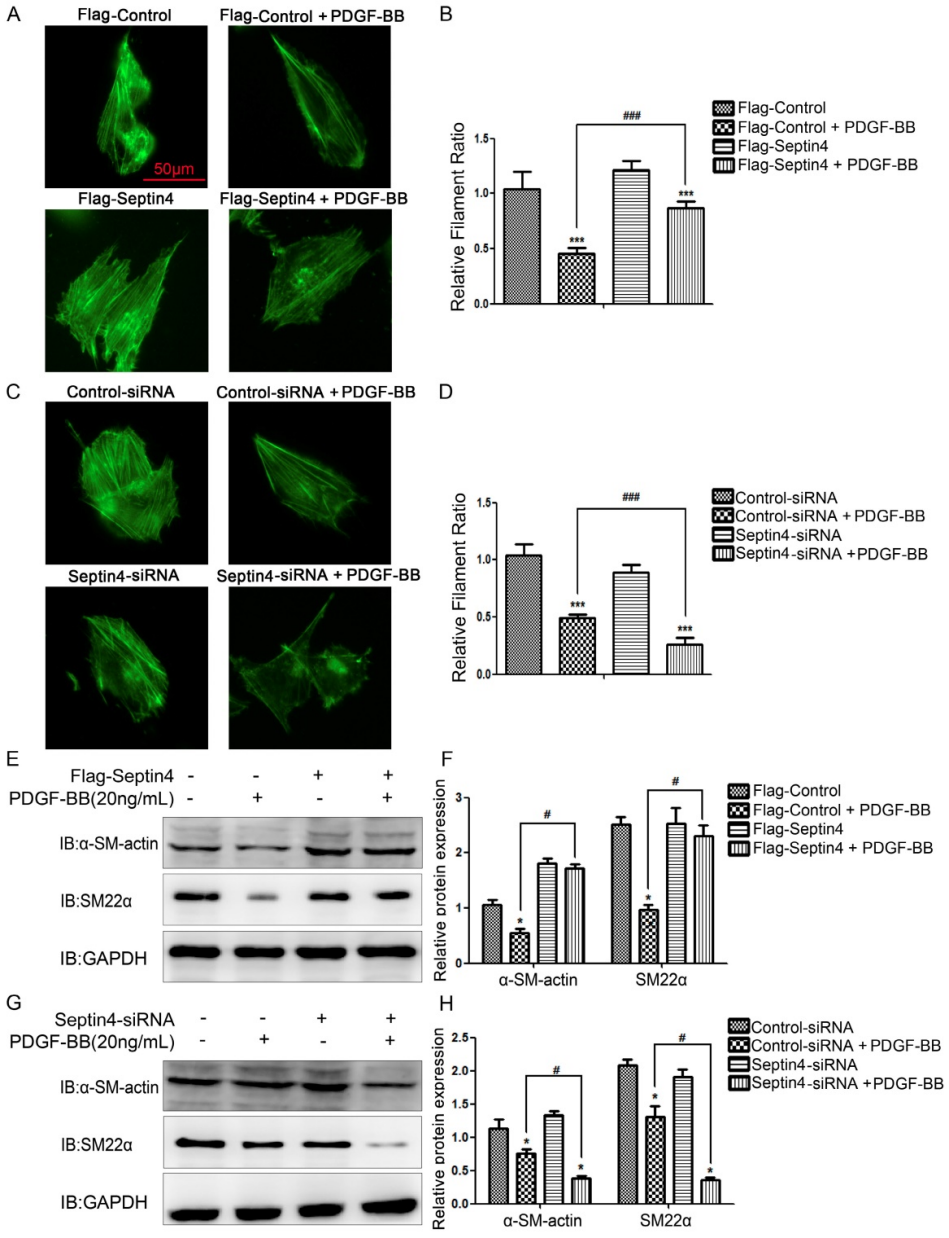
Septin4 resisted PDGF-BB-induced HAVSMCs phenotypic transformation. **(A)** HAVSMCs were transfected with the control or Flag-Septin4 plasmid for 36 hours, and treated with or without 20 ng/mL PDGF-BB for 24 hours. Phalloidine dye was used to assess HAVSMCs phenotypic transformation and intracellular myofilaments were labeled with green fluorescence; **(B)** quantitated data are mean ± SD (P<0.001).** (C-D)** As did HAVSMCs were transfected with the control-siRNA or Septin4-siRNA. **(E)** HAVSMCs were transfected with the control or Flag-Septin4 plasmid for 36 hours, and treated with or without 20 ng/mL PDGF-BB for 24 hours. α-SM-actin and SM22α were detected by Western-blot; **(F)** quantitated data are mean ± SD (P<0.001).** (G-H)** As did HAVSMCs were transfected with the control-siRNA or Septin4-siRNA.

In order to further clarify role of Septin4 in HAVSMCs phenotypic transformation, overexpression and knockdown Septin4 were performed in PDGF-BB-induced HAVSMCs phenotypic transformation. FITC-phalloidin showed that overexpression of Septin4 significantly antagonized PDGF-BB-induced HAVSMCs phenotypic transformation (filament ratio increased 89.7%; P<0.001) (*Figure 3A-B*). While, knockdown of Septin4 had the opposite effects (filament ratio decreased 46.6%; P<0.001) (*Figure 3C-D*).

In addition, overexpression of Septin4 significantly stabilized the expression of PDGF-BB-induced HAVSMCs contraction phenotype makers α-SM-actin and SM22α (*Figure 3E-F*). While, knockdown of Septin4 further reduced the expression of PDGF-BB-induced α-SM-actin and SM22α (*Figure 3G-H*).

The above results suggested that Septin4 may be a novel regulatory protein against phenotypic transformation of HAVSMCs.

### Septin4 was a novel interacting protein of STAT3 and SIRT1, forming a complex with SIRT1-STAT3, ensuing promoting the interaction between SIRT1 and STAT3

**Figure 4:**
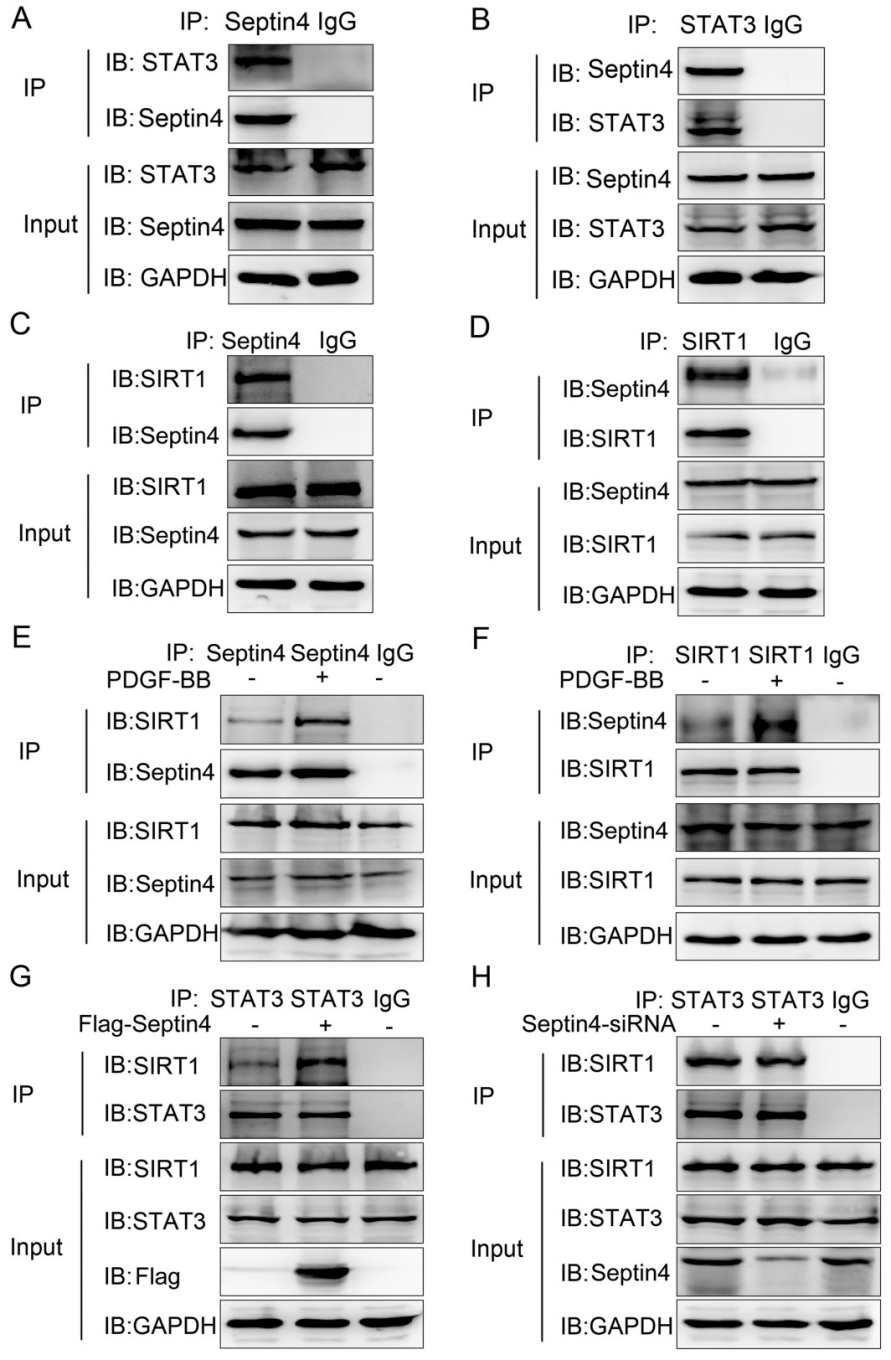
Septin4 formed a complex with SIRT1-STAT3, promoting the interaction between SIRT1 and STAT3. **(A-B)** In HAVSMCs, endogenous co-immunoprecipitation was performed to assess the interaction between Septin4 and STAT3. **(C-D)** As did the interaction between Septin4 and SIRT1. **(E-F)** Endogenous co-immunoprecipitation was performed to assess the interaction between Septin4 and SIRT1 with the addition of 20 ng/mL PDGF-BB.** (G)** HAVSMCs were transfected with the control or Flag-Septin4 plasmid for 36 hours. Endogenous co-immunoprecipitation between SIRT1 and STAT3 was assessed. **(H)** As did HAVSMCs were transfected with the control-siRNA or Septin4-siRNA.

To further explored the molecular mechanism of Septin4-regulated PDGF-BB-induced HAVSMCs proliferation, migration and phenotypic transformation, co-immunoprecipitation assays were performed to determine the interacting proteins of Septin4 in HAVSMCs. The results showed that Septin4 is a novel interacting protein of STAT3 (*Figure 4A-B*) and SIRT1 (*Figure 4C-D*) by endogenous co-immunoprecipitation assays. In addition, the interaction between Septin4 and SIRT1 was significantly enhanced in PDGF-BB-induced HAVSMCs (*Figure 4E-F*).

As SIRT1 and STAT3 play important roles in HAVSMCs proliferation, migration and phenotype transformation 13-18, 21-23, we further confirmed whether Septin4 participates in HAVSMCs proliferation, migration and phenotype transformation by regulating the interaction of SIRT1-STAT3. Co-immunoprecipitation assays results showed that overexpression of Septin4 significantly enhanced the interaction between SIRT1 and STAT3 (*Figure 4G*), while knockdown Septin4 significantly decreased the interaction between SIRT1 and STAT3 (*Figure 4H*).

The above results suggested that Septin4 forms a complex with SIRT1-STAT3 (Septin4, SIRT1 and STAT3 interact with each other), promoting the interaction between SIRT1 and STAT3.

### Septin4 promoted SIRT1-regulated STAT3-K685 deacetylation and STAT3-Y705 dephosphorylation in PDGF-BB-induced HAVSMCs model

**Figure 5:**
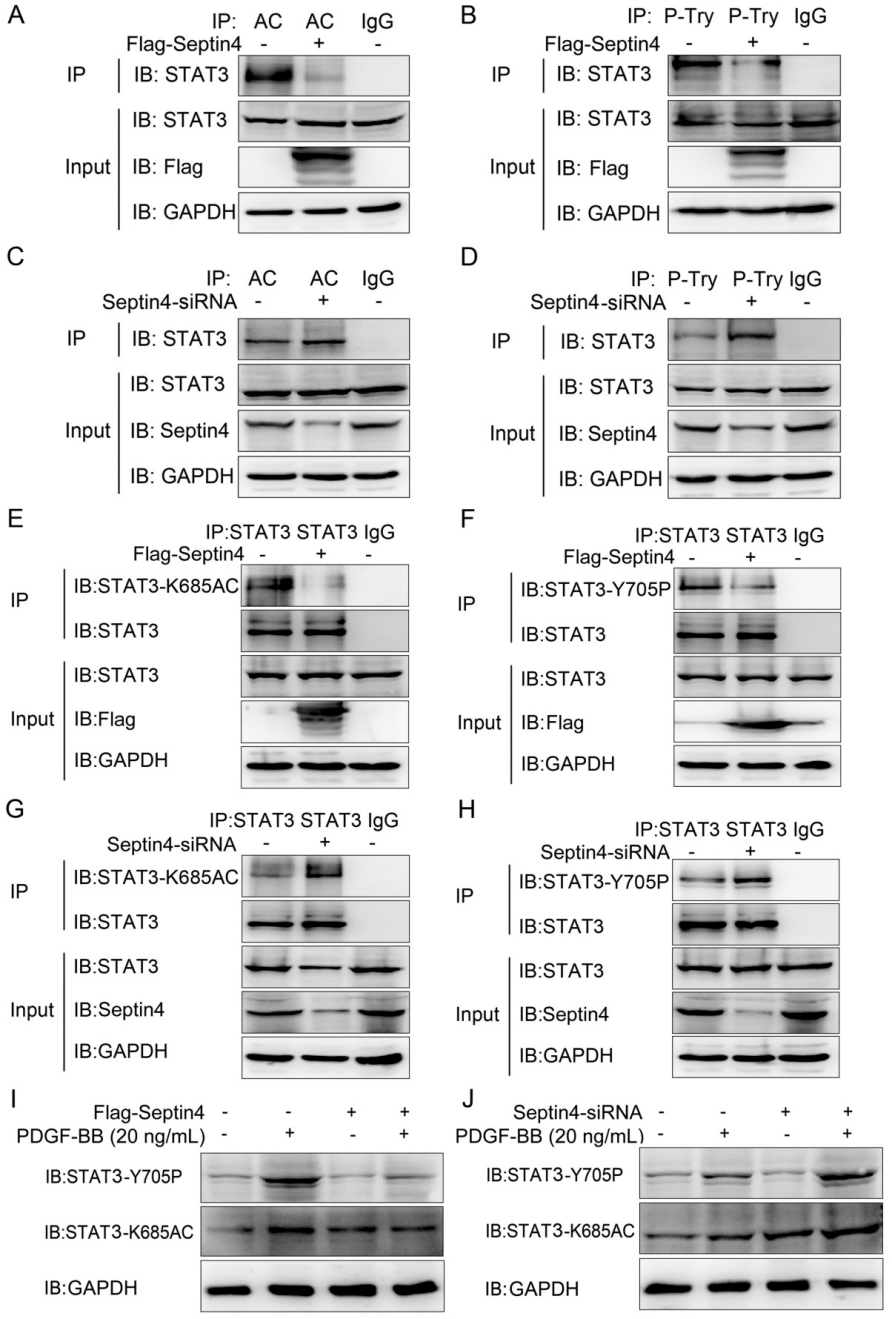
Septin4 promoted SIRT1-regulated STAT3-K685 deacetylation and STAT3-Y705 dephosphorylation in PDGF-BB-induced HAVSMCs model.** (A-B)** HAVSMCs were transfected with the control or Flag-Septin4 plasmid for 36 hours. Pan-acetylation or Pan-Tyr was isolated by immunoprecipitation, and STAT3 acetylation or phosphorylation level was assessed with anti-STAT3 antibody. **(C-D)** As did HAVSMCs were transfected with the control-siRNA or Septin4-siRNA. **(E-F)** HAVSMCs were transfected with the control or Flag-Septin4 plasmid for 36 hours. STAT3 was isolated by immunoprecipitation, and STAT3-K685 acetylation or STAT3-Y705 phosphorylation level was assessed with anti-STAT3-K685 antibody or STAT3-Y705 antibody. **(G-H)** As did HAVSMCs were transfected with the control-siRNA or Septin4-siRNA. **(I)** HAVSMCs were transfected with the control or Flag-Septin4 plasmid for 36 hours, with or without treatment of 20 ng/mL PDGF-BB. STAT3-K685 acetylation and STAT3-Y705 phosphorylation levels were assessed with anti-STAT3-K685 and anti-STAT3-Y705 antibodies. **(J)** As did HAVSMCs were transfected with the control-siRNA or Septin4-siRNA.

As STAT3 acetylation and phosphorylation were the key to promote HAVSMCs proliferation, migration and phenotypic transformation 13-18. And SIRT1 inhibited HAVSMCs proliferation, migration and phenotypic transformation 21-23. Therefore, we further clarify whether Septin4 regulates the acetylation and phosphorylation of STAT3 by enhancing the interaction between SIRT1 and STAT3. Co-immunoprecipitation assays results showed that overexpression of Septin4 significantly decreased the level of STAT3 acetylation and phosphorylation (*Figure 5A-B*), while knockdown Septin4 significantly enhanced the level of STAT3 acetylation and phosphorylation (*Figure 5C-D*).

Previous studies have shown that SIRT1 regulates acetylation and phosphorylation of STAT3 mainly acting on STAT3-K685 and STAT3-Y705, respectively 20. Therefore, in order to provide the most direct and important evidence that Septin4 promotes SIRT1-regulated STAT3 acetylation and phosphorylation, we further determine the sites of Septin4-inhibited STAT3 acetylation and phosphorylation. Co-immunoprecipitation assays results showed that overexpression of Septin4 significantly decreased the level of STAT3-K685 acetylation and STAT3-Y705 phosphorylation (*Figure 5E-F*), while knockdown Septin4 significantly enhanced the level of STAT3-K685 acetylation and STAT3-Y705 phosphorylation (*Figure 5G-H*).

Finally, we validated this mechanism in the PDGF-BB-induced HAVSMCs model. The results showed that overexpression of Septin4 significantly alleviated PDGF-BB-induced expression of STAT3-K685 acetylation and STAT3-Y705 phosphorylation (*Figure 5I*). While, knockdown of Septin4 had the opposite effects (*Figure 5J*).

Taken altogether, our graphical abstract reveals that Septin4 formed a complex with SIRT1-STAT3, enhancing the interaction between SIRT1 and STAT3, ensuing promoting SIRT1-regulated STAT3-K685 acetylation and STAT3-Y705 phosphorylation reductions, which inhibited PDGF-BB-induced HAVSMCs proliferation, migration and phenotype transformation.

## Discussion

Atherosclerosis is mainly caused by the proliferation and migration of HAVSMCs, and the phenotype transformation is the core to control the proliferation and migration of HAVSMCs 2-6. Recently, post-translational modification of protein is considered as the key in controlling the proliferation and migration of HAVSMCs, which provides new thoughts for the therapeutic strategies of atherosclerosis 2-6. Our study has the following major novel findings: **1,** expression of Septin4 were markedly increased in Apoe^-/-^ atherosclerosis mice and PDGF-BB-induced HAVSMCs. Knockdown of Septin4 significantly increased PDGF-BB-induced HAVSMCs proliferation, migration and phenotypic transformation, while overexpression of Septin4 had the opposite effects. **2,** Septin4 was a novel interacting protein of STAT3 and SIRT1, which formed a complex with SIRT1-STAT3, ensuing promoting for the interaction between SIRT1 and STAT3. **3,** STAT3-K685 acetylation and STAT3-Y705 phosphorylation are of critical importance in the regulation of STAT3 by Septin4 during atherosclerosis. Our study firstly identified the role of Septin4 and the mechanism of Septin4-SIRT1-STAT3 complex in the proliferation, migration and phenotypic transformation of HAVSMCs, providing new ideas for the therapeutic strategies of atherosclerosis.

Septin4 was considered as an apoptosis-related protein, playing an important role in the process of various organ damage 24-28. Septin4 localizes in the mitochondria and translocates to the nucleus upon pro-apoptotic stimuli, such as arabinoside, etoposide, staurosporine and Fas 28. Septin4 isform2 as the pro-apoptotic protein ARTS, the P-loop of ARTS is sufficient to induce apoptosis through activation of caspases 24-28. Our study found that Septin4 inhibited PDGF-BB-induced excessive proliferation and migration of HAVSMCs, which further improves the key role of Septin4 in the fight against abnormal proliferation and migration of cells.

SIRT1 and STAT3 play key roles in the proliferation and migration of HAVSMCs 21-23. SIRT1 decreases STAT3 phosphorylation by deacetylating STAT3 19-20. Activation of STAT3 phosphorylation significantly promoted proliferation and migration of HAVSMCs and STAT3 knockout remarkably reduced this phenomenon 14-18. However, the regulatory mechanism of SIRT1-STAT3 in HAVSMCs is still unclear. Our study found that Septin4 is a novel interacting protein of STAT3 and SIRT1, forming a complex with SIRT1-STAT3, ensuing promoting the interaction between SIRT1 and STAT3. In addition, Septin4 promotes SIRT1-regulated STAT3-K685 deacetylation and STAT3-Y705 dephosphorylation, inhibiting PDGF-BB-induced proliferation and migration of HAVSMCs.

Our study firstly clarified the key role of Septin4 in inhibiting proliferation, migration and phenotype transformation of HAVSMCs by regulating SIRT1-STAT3, which provides a theoretical basis for exploring new therapeutic strategies for atherosclerosis. It is meaningful to construct the knockout and transgenic mice of Septin4 and to explore its role in atherosclerosis in the future. It will also be significant to explore the role and mechanism of Septin4 in other cardiovascular diseases.

In conclusion, Septin4 formed a complex with SIRT1-STAT3, enhancing the interaction between SIRT1 and STAT3, ensuing promoting SIRT1-regulated STAT3-K685 deacetylation and STAT3-Y705 dephosphorylation, which inhibited PDGF-BB-induced HAVSMCs proliferation, migration and phenotype transformation.

## Abbreviations

HAVSMCs: human aortic vascular smooth muscle cells
PDGF-BB: platelet-derived growth factor BB
PARP1: poly ADP-ribose polymerase 1
STAT3: signal transducer and activator of transcription 3
